# The Various Conditions under Which Albumen Is Found in the Urine

**Published:** 1881-12

**Authors:** 


### Original Translations.

### ARTICLE XV.

### THE VARIOUS CONDITIONS UNDER WHICH ALBUMEN IS FOUND IN THE URINE. By ED. ROBIN.

Urine becomes albuminous: 1. In croup or laryngeal diphtheria; in ascites, or far-advanced abdominal dropsies; in that emphysema in which much dyspnœa takes place; in pulmonary phthisis, especially if complicated with pneumonia, and accompanied by much difficulty in the respiration; in the child-bearing state, after the abdominal circulation is interfered with; that is, in those diseases in which a notable diminution of combustion (oxydation) follows a difficult respiration.

2. In cyanosis, whatever its cause, and in heart troubles carried to that extent that the patients are in a state of partial asphyxia; hence in cases in which an obstacle to the circulation, or a vicious conformation of the heart, prevents hæmatosis from being as complete and as rapid as under ordinary circumstances.

3. In eclampsia.

4. In cholera, a disease which diminishes considerably the oxydation of the blood.

5. In idiopathic or in traumatic lesion of nerve-centers, with lowering of temperature, and a notable diminution of combustion.

6. In diabetes, a disease in which, often enough, some primitive and analogous lesion seems to have existed; in which, besides, a great amount of sugar in the blood impedes the combustion of albuminous matter and hæmatosis; in which, finally, as Bouchardat observed, the temperature is lowered by one or two degrees in deeply diseased subjects.

7. In that sort of deficiency of nervous fluid which characterizes the state called curvature of the spine, which is most likely accompanied by a considerable diminution of calorification.

8. In the natural or artificial suppression of cutaneous perspiration, a suppression which sensibly diminishes the absorbing capacity of blood for oxygen, which may bring on a sort of semi-asphyxia, at any rate, a considerable lowering of temperature, which necessarily produces a marked diminution in the latent combustion of the tissues.

Thus urine becomes albuminous from the use of impermeable artificial coverings to the skin; and the same phenomena is not rare in those diseases, as measles, scarlet fever and small-pox, in which the functions of the skin are liable to be greatly interfered with.

From an analogous cause, urine is albuminous as a sequel of an intense cooling of the surface of the body from cold.

Lastly, Bright's disease, in which the urine is always found to be albuminous, is precisely referred to many of the causes just enumerated: heart disease; changes in the liver; a retardation or suspension of circulation in the abdomen, whatever the cause; a sudden impression of intense cold; large imbibition of alcoholics; pulmonary phthisis with dyspnœa, etc.

As a general rule, the urine of common quadrupeds, and that of birds, does not contain albumen. In reptiles, on the contrary, in batrachia, in frogs, at least, so remarkable on account of their low animal heat, some albumen is always passed with the urine (Dumas).

It is not quite demonstrated, yet very probable, that urine becomes albuminous under the influence of those agents which, according to one, protect to quite an extent from latent combustion. I have but few facts to substantiate that view.

Urine becomes albuminous in protracted anæsthesia, and in slow poisoning by cyanhydric acid; habitual drunkenness predisposes to albuminuria to quite an extent. This state suddenly appears sometimes in syphilitic patients under a course of mercury. . . . . The albumen discharged represents so much organic matter which has escaped a transformation into urea and uric acid, from a lack of activity in the oxydation of the blood.

Whatever the true theory, it remains a constant fact that *any important alteration in hæmatosis gives rise to albuminuria.*

The foregoing were condensed from a communication made in 1851 to the *Académie des Sciences*, by myself. The following are facts generally ascertained since :

*The fœtus of quadrupeds* resembles cold-blooded animals as to its hæmatosis, and its urine is albuminous, and remarkable for a total absence of urea.

When death is approaching, the respiratory movements and the cardiac contractions weaken to quite an extent ; the lungs become congested ; in a word, hæmatosis is considerably diminished, and, as observations prove, this state is liable to cause albuminuria. . . . . Septicæmia, hospital gangrene, phlegmonous erysipelas, yellow fever, typhoid, and typhus fever, which are all remarkable for a diminution of hæmatosis, are also remarkable for the frequency of albuminous urine. This condition of the urine is, indeed, one of the most constant symptoms of typhoid fever, and, according to Finger's observation, the renal tissue may remain intact.

From the same cause, miliary sweat, in which hæmatosis is so diminished and the blood so thin, is one of the diseases accompanied by albuminuria.

Jaundice often depends on a rapid diminution of hæmatosis, and it is sometimes accompanied by albuminous urine.

Chlorosis, a type of pathological anæmia, diminishes notably the number of red corpuscles, and often has albuminuria for one of its symptoms.

Gout; red gravel; rheumatism ; sicknesses in which hæmatosis is frequently diminished, are often accompanied by albuminuria.

Let the alteration result from tuberculosis, syphilis, cancer, glanders, malaria, scrofula, or other causes, all cachexias have this in common, that they diminish the formation of blood corpuscles, and diminish in a marked degree respiratory combustion. All of them diminish the tenacity of tissues, and end by allowing an escape of albumen with the urine.

In meningitis, the nature and the seat of the disease; the somnolence, coma, nausea, vomiting, convulsions ; all seem to indicate marked disturbance in hæmatosis. (See Book III of of my works on the Reformation of Medical Sciences, theory of purgatives and emetics). Indeed, according to Rosenstein, albumen appears in the urine from the beginning of the sickness, in adults as well as in children.

*Influence of cathartics and emetics.* According to my theory on the subject, quite a number of cathartics and emetics produce this action by diminishing more or less rapidly the formation of blood. Hence it would be quite natural for drastic cathartics to give rise to albuminuria; and this has often been observed. Nitrate of potassium itself is liable to render the urine albuminous, whether it is taken in repeated large doses, or in small doses for a long time.

*Influence of anœsthetics.* According to the same theory, anæsthetics protect from putrefaction after death, and retard blood formation during life. And, in accordance with what was already known of chloroform and several ethers, it is now ascertained that chloral hydrate is anti-putrid after death ; that it retards greatly vital combustion; that in repeated large doses it causes nausea and vomiting; that it brings on a relaxation of the tissues ; that long taken internally, it may bring on albuminuria, anasarca, and a sort of poisoning resembling that caused by ergot. (Smith, *Boston Med. and Surg. Journal*).

Coffee is antiseptic, sedative, not very energetic ; and according to my views, may, when taken in large doses, give a darker color to the blood, diminish the exhalation of carbon dioxide and the production of urea ; it may act as a diuretic, and let albumen escape with the urine. The same is true of fuchsine, especially arsenical fuchsine ; it is antiseptic, retards hæmatosis, and may be accompanied by albuminuria.

*Metallic compounds.* Those salts of the metals proper which do not absorb oxygen are generally antiseptic after death, and poisonous during life. In my view, the subsequent death, and the symptoms which precede it, manifest in various degrees an arrest of hæmatosis. All of them may doubtless cause albuminuria; at least, the number of them in which the fact has been ascertained has considerably increased since I first brought out my theory. Then my only observations referred to mercurials, but now the following are said to act thus: the soluble compounds of antimony and lead (Ollivier); some silver compounds (Lionville); salts of cadmium; nitrate of uranium; various copper salts; arsenical compounds; gold and palladium chlorides.

*Moral influences.* It is a well-known fact that sad moral affections may diminish hæmatosis to quite an extent. It is also a well-known fact that these may cause diuresis, intestinal evacuations, vomiting, albuminuria (Prout, etc.)

*Influence of water taken internally.* Upon injecting a certain amount of water into the veins, the blood becomes very thin, there is a marked decrease in hæmatosis, with a sedative effect very marked. As in the preceding cases, the marked arrest of function is accompanied by an escape of albumen into the urine (Magendie). These results have been verified by Hermann.

*Iufluence of oxydizing agents*—that is, agents which are known to diminish the quantity of free oxygen, and to rarefy the atmosphere. In phosphorus poisoning, an energetic oxydizing agent, the quantity of oxygen in the blood is markedly diminished. This diminution is otherwise manifested by a marked change in color, by a striking increase of fat, enlargement of the liver and vomiting. What becomes of the albumen of the blood under such circumstances? It passes away with the urine (C. Schultzen).

According to the experiments of Cl. Bernard, *carbon protoxide gas* forms with hæmoglobine a kind of compound more intimate than that resulting from oxygen, so that when it is respired, it has a tendency to displace this gas from the blood globules, and consequently diminish hæmatosis; the temperature is lowered, the amount of urea diminished, while that of uric acid is increased (Ritter). This also justifies my theory, for carbon protoxyde is antiseptic and preserves dead bodies, its inspiration also causes albuminuria. At high altitudes, a rarefaction of the air ought to diminish hæmatosis. Indeed, the blood turns darker, the liver often becomes congested, vomiting, as above described, becomes manifest if the transition is sudden, and urea is diminished in the urine (Bert). Also, albuminuria is much more frequent on high mountains than at the level of the sea (Jourdanet).

The preceding facts demonstrate in the highest degree the exactness of our theory, and their publication, we hope, will stimulate also to greater progress in that line.—*Revue Medical, July* 30, 1881.

---

LISTERISM TOTTERING.—Just as every body thought the carbolic spray of Lister had become an established method in surgery, it receives a stunning blow from one of its former advocates, Mr. Keith, the great ovariotomist. At the International Congress, according to a letter in the *Boston Medical Journal*, Prof. Keith declared, the subject being under discussion, that he had abandoned the antiseptic treatment altogether. "True," he said, "I had eighty successive recoveries under Lister's method, and *stopping there* it would be a wonderful showing. *But out of the next twenty-five I lost seven.* One died of acute septicæmia, in spite of the most thorough antiseptic precaution; three of unquestionable carbolic acid poisoning; one of renal hæmorrhage." He went on to say that out of the eighty consecutive cases (or rather he said it first) many came too near dying; that a large number got a high temperature—105,° 106,° 107° Fahrenheit—the evening following the operation, but he said "they happened to pull through." He then said that since he had for four months past abandoned the antiseptic method, and relied upon perfect cleanliness, care in controlling hæmorrhage, and thorough drainage, his cases were giving him much less trouble, and he was getting more satisfactory results.

He now stopped for a few moments, hesitating, as he must have realized the importance of his words, knowing that the whole world—surgical—was lending a "listening ear" to his utterance. The silence was "audible." Then he raised his head, and looking his audience squarely in the face, he said: "Gentlemen, I have felt it my duty to make these statements, for *they are true*," and took his seat. I shall not attempt to describe the applause nor the effect of his statements. Prof. Keith, by the way, told me privately that he almost died himself from using the carbolic acid so much. He got renal hæmorrhage and debility to an alarming degree.—*Pacific Med. and Surg. Journal.*

